# Supplementary material for: Multi‐dataset identification of innovative feature genes and molecular mechanisms in keratoconus
Source: J Cell Mol Med. 2024 Sep 19;28(18):e70079. doi: 10.1111/jcmm.70079 (PMC11412914; doi:10.1111/jcmm.70079)
Supplement: Supplementary file 2 — Material S2. [file JCMM-28-e70079-s002.zip › 7.GSEA analysis/6.SNORA74B/KEGG_FC_GAMMA_R_MEDIATED_PHAGOCYTOSIS.html]

Details for gene set KEGG\_FC\_GAMMA\_R\_MEDIATED\_PHAGOCYTOSIS[GSEA]

|  || Dataset | SNORA74B.group.cls#h\_versus\_l |
| Phenotype | group.cls#h\_versus\_l |
| Upregulated in class | l |
| GeneSet | KEGG\_FC\_GAMMA\_R\_MEDIATED\_PHAGOCYTOSIS |
| Enrichment Score (ES) | -0.41551784 |
| Normalized Enrichment Score (NES) | -1.7085212 |
| Nominal p-value | 0.004056795 |
| FDR q-value | 0.41028446 |
| FWER p-Value | 0.361 |
Table: GSEA Results Summary

  

Fig 1: Enrichment plot: KEGG\_FC\_GAMMA\_R\_MEDIATED\_PHAGOCYTOSIS      
 Profile of the Running ES Score & Positions of GeneSet Members on the Rank Ordered List

  

| SYMBOL | TITLE | RANK IN GENE LIST | RANK METRIC SCORE | RUNNING ES | CORE ENRICHMENT || 1 | PIK3R5 | na | 170 | 0.800 | 0.0498 | No |
| 2 | PLA2G4D | na | 2149 | 0.358 | 0.0172 | No |
| 3 | PRKCG | na | 2539 | 0.330 | 0.0286 | No |
| 4 | HCK | na | 3040 | 0.298 | 0.0346 | No |
| 5 | PLA2G4E | na | 5383 | 0.212 | -0.0185 | No |
| 6 | INPP5D | na | 6064 | 0.190 | -0.0251 | No |
| 7 | ASAP3 | na | 6075 | 0.190 | -0.0124 | No |
| 8 | PIK3R1 | na | 8116 | 0.133 | -0.0622 | No |
| 9 | ARPC4 | na | 8193 | 0.131 | -0.0554 | No |
| 10 | PLCG2 | na | 8239 | 0.131 | -0.0478 | No |
| 11 | FCGR1A | na | 8319 | 0.129 | -0.0413 | No |
| 12 | FCGR2A | na | 8572 | 0.123 | -0.0401 | No |
| 13 | AKT2 | na | 9380 | 0.105 | -0.0562 | No |
| 14 | PLCG1 | na | 9672 | 0.099 | -0.0578 | No |
| 15 | VAV2 | na | 10064 | 0.091 | -0.0629 | No |
| 16 | GSN | na | 10144 | 0.090 | -0.0590 | No |
| 17 | LIMK1 | na | 10417 | 0.085 | -0.0611 | No |
| 18 | PIKFYVE | na | 10605 | 0.081 | -0.0609 | No |
| 19 | SPHK1 | na | 10715 | 0.080 | -0.0586 | No |
| 20 | MYO10 | na | 10906 | 0.076 | -0.0589 | No |
| 21 | PIP4K2B | na | 11358 | 0.069 | -0.0672 | No |
| 22 | DNM2 | na | 12643 | 0.051 | -0.1007 | No |
| 23 | VASP | na | 12788 | 0.049 | -0.1015 | No |
| 24 | RPS6KB2 | na | 12925 | 0.048 | -0.1022 | No |
| 25 | PIK3R2 | na | 12946 | 0.047 | -0.0995 | No |
| 26 | PIK3CA | na | 13079 | 0.046 | -0.1001 | No |
| 27 | DNM3 | na | 13211 | 0.045 | -0.1009 | No |
| 28 | RPS6KB1 | na | 13499 | 0.042 | -0.1063 | No |
| 29 | PLA2G4F | na | 13561 | 0.041 | -0.1053 | No |
| 30 | DNM1L | na | 14208 | 0.034 | -0.1216 | No |
| 31 | ARPC3 | na | 14918 | 0.025 | -0.1404 | No |
| 32 | ARPC5L | na | 15295 | 0.022 | -0.1498 | No |
| 33 | PIK3CB | na | 15769 | 0.017 | -0.1623 | No |
| 34 | VAV3 | na | 16097 | 0.014 | -0.1708 | No |
| 35 | PLA2G6 | na | 16344 | 0.011 | -0.1771 | No |
| 36 | WAS | na | 16570 | 0.009 | -0.1830 | No |
| 37 | PLD2 | na | 16641 | 0.008 | -0.1845 | No |
| 38 | MAPK3 | na | 16753 | 0.007 | -0.1872 | No |
| 39 | PRKCD | na | 17017 | 0.005 | -0.1945 | No |
| 40 | WASF2 | na | 17499 | -0.001 | -0.2083 | No |
| 41 | WASL | na | 17732 | -0.003 | -0.2148 | No |
| 42 | ARPC2 | na | 17775 | -0.003 | -0.2158 | No |
| 43 | CFL1 | na | 17798 | -0.004 | -0.2162 | No |
| 44 | ARPC5 | na | 17932 | -0.005 | -0.2197 | No |
| 45 | PTPRC | na | 18235 | -0.009 | -0.2278 | No |
| 46 | AKT1 | na | 18366 | -0.010 | -0.2309 | No |
| 47 | ARPC1A | na | 18393 | -0.011 | -0.2309 | No |
| 48 | PIP5K1C | na | 18480 | -0.012 | -0.2326 | No |
| 49 | PLD1 | na | 18510 | -0.012 | -0.2326 | No |
| 50 | CDC42 | na | 18770 | -0.015 | -0.2391 | No |
| 51 | PIK3CG | na | 18884 | -0.016 | -0.2413 | No |
| 52 | RAC2 | na | 18892 | -0.016 | -0.2404 | No |
| 53 | SYK | na | 19828 | -0.027 | -0.2655 | No |
| 54 | RAC1 | na | 19881 | -0.027 | -0.2652 | No |
| 55 | PIK3CD | na | 20354 | -0.032 | -0.2766 | No |
| 56 | MAPK1 | na | 20606 | -0.035 | -0.2815 | No |
| 57 | LAT | na | 20912 | -0.039 | -0.2876 | No |
| 58 | RAF1 | na | 21074 | -0.041 | -0.2895 | No |
| 59 | FCGR2B | na | 21581 | -0.047 | -0.3009 | No |
| 60 | PRKCA | na | 22239 | -0.055 | -0.3161 | No |
| 61 | WASF1 | na | 22378 | -0.057 | -0.3162 | No |
| 62 | ARF6 | na | 22549 | -0.059 | -0.3171 | No |
| 63 | PLA2G4A | na | 24085 | -0.082 | -0.3558 | No |
| 64 | DNM1 | na | 24199 | -0.084 | -0.3533 | No |
| 65 | LIMK2 | na | 24245 | -0.085 | -0.3488 | No |
| 66 | MARCKS | na | 25648 | -0.110 | -0.3818 | No |
| 67 | ARPC1B | na | 26427 | -0.126 | -0.3956 | No |
| 68 | ASAP2 | na | 26620 | -0.131 | -0.3923 | No |
| 69 | CRK | na | 26740 | -0.134 | -0.3866 | No |
| 70 | SCIN | na | 27047 | -0.140 | -0.3858 | No |
| 71 | NCF1 | na | 27684 | -0.156 | -0.3935 | No |
| 72 | WASF3 | na | 28101 | -0.167 | -0.3940 | No |
| 73 | PAK1 | na | 28478 | -0.179 | -0.3927 | No |
| 74 | CRKL | na | 29271 | -0.203 | -0.4016 | Yes |
| 75 | LYN | na | 29275 | -0.203 | -0.3878 | Yes |
| 76 | PIK3R3 | na | 29453 | -0.208 | -0.3787 | Yes |
| 77 | MARCKSL1 | na | 29873 | -0.221 | -0.3757 | Yes |
| 78 | FCGR3A | na | 29886 | -0.221 | -0.3609 | Yes |
| 79 | SPHK2 | na | 30334 | -0.238 | -0.3575 | Yes |
| 80 | FCGR2C | na | 31076 | -0.272 | -0.3603 | Yes |
| 81 | MAP2K1 | na | 31129 | -0.275 | -0.3430 | Yes |
| 82 | GAB2 | na | 32350 | -0.344 | -0.3547 | Yes |
| 83 | VAV1 | na | 32928 | -0.388 | -0.3448 | Yes |
| 84 | PIP5K1A | na | 33089 | -0.403 | -0.3219 | Yes |
| 85 | CFL2 | na | 33537 | -0.454 | -0.3037 | Yes |
| 86 | DOCK2 | na | 33838 | -0.501 | -0.2781 | Yes |
| 87 | PRKCB | na | 33882 | -0.508 | -0.2445 | Yes |
| 88 | ASAP1 | na | 33924 | -0.518 | -0.2103 | Yes |
| 89 | AMPH | na | 34115 | -0.560 | -0.1775 | Yes |
| 90 | PRKCE | na | 34306 | -0.615 | -0.1409 | Yes |
| 91 | AKT3 | na | 34657 | -0.880 | -0.0908 | Yes |
| 92 | PIP5K1B | na | 34724 | -1.356 | 0.0001 | Yes |
Table: GSEA details [plain text format]

  

Fig 2: KEGG\_FC\_GAMMA\_R\_MEDIATED\_PHAGOCYTOSIS      
 Blue-Pink O' Gram in the Space of the Analyzed GeneSet

  

Fig 3: KEGG\_FC\_GAMMA\_R\_MEDIATED\_PHAGOCYTOSIS: Random ES distribution      
 Gene set null distribution of ES for **KEGG\_FC\_GAMMA\_R\_MEDIATED\_PHAGOCYTOSIS**
